# Fluorescent magnetic nanoparticle-labeled mesenchymal stem cells for targeted imaging and hyperthermia therapy of *in vivo* gastric cancer

**DOI:** 10.1186/1556-276X-7-309

**Published:** 2012-06-18

**Authors:** Jing Ruan, Jiajia Ji, Hua Song, Qirong Qian, Kan Wang, Can Wang, Daxiang Cui

**Affiliations:** 1 Department of Bio-Nano Science and Engineering, Key Laboratory for Thin Film and Microfabrication of Ministry of Education, Institute of Micro-Nano Science and Technology, Shanghai Jiao Tong University, 800 Dongchuan Road, Shanghai, 200240, People's Republic of China; 2 Department of Orthopedics, Changzheng Hospital affiliated to Second Military Medical University, 451Fengyang Road, Shanghai, 200003, People's Republic of China

**Keywords:** fluorescent magnetic nanoparticle, mesenchymal stem cells, gastric cancer, targeted imaging, hyperthermia therapy

## Abstract

How to find early gastric cancer cells *in vivo* is a great challenge for the diagnosis and therapy of gastric cancer. This study is aimed at investigating the feasibility of using fluorescent magnetic nanoparticle (FMNP)-labeled mesenchymal stem cells (MSCs) to realize targeted imaging and hyperthermia therapy of *in vivo* gastric cancer. The primary cultured mouse marrow MSCs were labeled with amino-modified FMNPs then intravenously injected into mouse model with subcutaneous gastric tumor, and then, the *in vivo* distribution of FMNP-labeled MSCs was observed by using fluorescence imaging system and magnetic resonance imaging system. After FMNP-labeled MSCs arrived in local tumor tissues, subcutaneous tumor tissues in nude mice were treated under external alternating magnetic field. The possible mechanism of MSCs targeting gastric cancer was investigated by using a micro-multiwell chemotaxis chamber assay. Results show that MSCs were labeled with FMNPs efficiently and kept stable fluorescent signal and magnetic properties within 14 days, FMNP-labeled MSCs could target and image *in vivo* gastric cancer cells after being intravenously injected for 14 days, FMNP-labeled MSCs could significantly inhibit the growth of *in vivo* gastric cancer because of hyperthermia effects, and CCL19/CCR7 and CXCL12/CXCR4 axis loops may play key roles in the targeting of MSCs to *in vivo* gastric cancer. In conclusion, FMNP-labeled MSCs could target *in vivo* gastric cancer cells and have great potential in applications such as imaging, diagnosis, and hyperthermia therapy of early gastric cancer in the near future.

## Background

Gastric cancer is currently the second most common cancer and third most common cause of cancer-related death in China [1,2], and it is still the second most common cause of cancer-related death in the world [3]. Gastric cancer remains difficult to cure because most patients present with advanced disease [4]. Therefore, how to recognize, track, and kill early gastric cancer cells is still one key scientific problem for early diagnosis and therapy of patients with gastric cancer.

Since 1998, we have been trying to establish an early gastric cancer prewarning system [5,6]. In order to recognize early gastric cancer cells, we selected potential biomarkers associated with gastric cancer, combined nanoparticles and molecular imaging techniques, and tried to identify early gastric cancer cells *in vivo*[7-15]. Although some differently expressed genes associated with gastric cancer were identified [16,17], no gene could be confirmed as a specific biomarker of gastric cancer. Therefore, looking for a novel pathway to recognize and treat early gastric cancer cells *in vivo* has become our major concern.

Stem cell therapy is one emerging potential therapeutic method for cancer therapy following the operation, chemotherapy, and radiotherapy. Mesenchymal stem cells (MSCs) are a subset of nonhematopoietic multipotent cells found primarily within the bone marrow stroma. As one kind of promising seed cells on cancer therapy, MSCs not only have self-renewing and mutlipotent features but can also efficiently carry and deliver genes into a specific location [18-24], have immunomodulatory property, and can home to the sites of active tumorgenesis [25-28]. Therefore, it is possible to use MSCs to target and identify gastric cancer cells *in vivo*. Furthermore, the combination of nanotechnology and MSCs exhibits great potential in the diagnosis and therapy of early gastric cancer. Up to date, no report fully confirms that MSCs could target imaging and treat gastric cancer.

Herein, we labeled the mouse marrow MSCs with amino-modified fluorescent magnetic nanoparticles (FMNPs) and injected FMNP-labeled MSCs into the mouse model with subcutaneous gastric cancer from the mouse gastric cancer cell line mouse forestomach carcinoma (MFC) cells via the tail vein. Then, we investigated the distribution and targeting ability of the labeled MSCs in nude mice by fluorescence imaging system and magnetic resonance imaging (MRI) system. Then, we irradiated subcutaneous gastric cancer tissues in nude mice by a given external magnetic field and finally explored the possible mechanism of MSCs migrating to *in vivo* gastric cancer cells. Results showed that FMNP-labeled MSCs could target and recognize *in vivo* gastric cancer cells and could inhibit the growth of gastric cancer cells under the given external magnetic field. Therefore, FMNP-labeled MSCs have great potential in applications such as targeted imaging and simultaneous therapy of early gastric cancer in the near future.

## Methods

All animal experiments (NO.SYXK2007-0025) were approved by the Institutional Animal Care and Use Committee of Shanghai Jiao Tong University.

### Primary culture and identification of mouse MSCs

MSCs were isolated according to a protocol [29] and were cultured with Dulbecco's modified Eagle's medium (DMEM; Gibco, Shanghai, China) with 20% fetal bovine serum (FBS; Hyclone, Thermo Scientific, Logan, UT, USA), 100 U/mL penicillin, and 100 mg/mL streptomycin (Gibco) at 37 °C in a 5% CO_2_ incubator. MSC medium was changed once every 2 days. In order to identify MSCs, passage 3 MSCs were fixed with 4% paraformaldehyde, stained with R-phycoerythrin (PE)-conjugated CD90 antibody (BioLegend, San Diego, CA, USA) and fluorescein isothiocyanate (FITC)-conjugated CD29 antibody (BioLegend), respectively, and observed with a laser confocal scanning microscope (Leica TCS SP5, Leica Microsystems, Shanghai, China). Passage 4 MSCs were detached with 0.05% EDTA in 0.1% phosphate buffered saline (PBS) and rinsed with 0.1% PBS. And then, PE-conjugated CD90 monoclonal antibody, FITC-conjugated CD29 monoclonal antibody, and PE-conjugated CD45 monoclonal antibody were respectively added into cells with 0.1% PBS containing 0.5% BSA (pH 7.2) and incubated at 4 °C for 30 min. Cells were rinsed in 0.1% frozen PBS and observed by a Calibur flow cytometer (Becton-Dickinson, Franklin Lakes, NJ, USA).

MSCs were further characterized by differentiation assays. Passage 3 MSCs were seeded on the 24-well culture plates at a density of 5 × 10^4^/well and incubated at 37 °C in an incubator with 5% CO_2_. After 24 h, when the confluence of MSCs reached 80%, MSCs were cultured with different kinds of differentiation medium. Osteoblast differentiation culture medium consists of DMEM with 0.1 μmol/L dexamethasone (Sigma-Aldrich, Shanghai, China), 10 mmol/L β-sodium glycerophosphate (Sigma), 50 μmol/L ascorbic acid (Sigma), 100 U/L penicillin-streptomycin, and 10% FBS. Adipocyte differentiation medium consists of DMEM with 10 mg/L insulin (Sigma), 1 μmol/L dexamethasone, 0.5 mmol/L 3-isobutyl-1-methylxanthine (Sigma), and 100 μmol/L indomethacin (Sigma). Chondrocyte differentiation culture medium consists of DMEM with 50 μg/mL ascorbic acid, 40 μg/mL proline (Sigma), 1% insulin-transferrin-selenium (Sigma), 0.1 μM dexamethasone (Sigma), and 10 ng/ml TGF-β (Sigma). Three weeks later, differentiated osteoblasts were stained with alkaline phosphatase (Sigma), differentiated adipocytes were stained with oil red O (Sigma), and differentiated chondrocytes were identified with toluidine blue stain (Ameresco, Solon, OH, USA).

### Preparation of FMNP-labeled MSCs

Silica-coated FMNPs were synthesized and characterized according to our previous reports [30,31]. Ethanol (95 mL) and 2 mL 3-aminopropyltriethoxysilane (APS) were added to form a mixed solution and allowed to react at room temperature for 24 h. The amino-modified FMNPs were separated by permanent magnet and were washed with deionized water three times then saved for further usage. Prepared amino-modified FMNPs were characterized by a transmission electron microscope (TEM). The fluorescent and magnetic properties of the amino-modified FMNPs were characterized by using the photoluminescence (PL) spectra (Perkin Elmer LS 55 spectrofluorimeter, PerkinElmer, Waltham, MA, USA) and superconducting quantum interference device magnetometer (PPMS-9 T, Quantum Design, Beijing, China). Zeta-potential value of amino-modified FMNPs was measured with particle sizing systems (NICOMP 380 ZLS, PSS, Port Richey, FL, USA). Then MSCs were treated with medium containing amino-modified FMNPs (50 μg/mL) for 4 h. Afterward, the cells were stained with Prussian blue and visualized under a light microscope (Olympus IX71, Olympus, Shanghai, China); the labeled MSCs were also stained with 1 mM Hoechst 33258 in PBS (pH 7.4) for 5 min, and fluorescent signal of MSCs was observed by a laser confocal scanning microscope with excitation wavelength of 488 nm (Leica TCS SP5). The labeled MSCs were also embedded with epoxy resin and made into ultra-thin slices and finally observed with a transmission electronic microscope (JEOL JEM2010, JEOL Co. Ltd., Shanghai, China).

In order to confirm whether the MSCs could be labeled up to 14 days, we collected unlabeled MSCs and labeled MSCs at 7 and 14 days into eppendorf tubes to perform the MR imaging. We also examined fluorescence signal of MSCs by a fluorescence microscope.

### Cell viability assay

The effect of amino-modified FMNPs on MSCs was evaluated by using the Cell Counting Kit-8 (CCK8) assay. MSCs in 96-well plates (5,000 cells per well) were incubated with MSC medium containing 50 μg/mL of amino-modified FMNPs for 4 h at 37 °C. After replacing with fresh medium, the MSCs continued to culture for 1 to 7 days. The OD values of the cells were measured by using the Multiskan mircoplate reader (Thermo MK3, Thermo Scientific) according to the protocol of the CCK8 assay kit, and the survival rate of the cells was calculated according to the following equation: Cell viability (%) = optical density (OD) of the treated cells/OD of the untreated cells × 100.

### Fluorescence imaging and MRI of gastric cancer cells *in vivo*

All animal experiments complied with the local ethics committee. MFC cells (5 × 10^6^) were injected subcutaneously into the right fore of the nude mice ages 6 to 8 weeks old. When tumors grew to a diameter of approximately 5 mm, 5 × 10^6^ MSCs labeled with FMNPs were intravenously injected into the mouse models with gastric cancer (*n* = 3) via the tail vein. In the control experiment, FMNPs were intravenously injected into nude mouse models (*n* = 3) loaded with gastric cancer. These mice were imaged at 7 and 14 days post-injection by using IVIS Lumina imaging system (Xenogen (Caliper Life Sciences), Hopkinton, MA, USA), and MR imaging was performed at 3, 7, 10, and 14 days after post-injection by using GE HDX 3.0 T MR imaging instrument (GE Healthcare, Chalfont St. Giles, UK). The fluorescence signals were acquired at a lateral position on the condition of 465-nm excitation filter and DsRed emission filter. Magnetic resonance signals were obtained with coronal and transected T2-weighted spin echo pulse sequences, and the following imaging parameters were used: TR = 2,500 ms, TE = 80 to approximately 90 ms, FOV = 40 mm, NEX = 2, and slice thickness = 2.0 mm. Then, these mice were killed. Then major organs, such as the liver, heart, lung, brain, and kidney and the tumor, were collected to perform further fluorescence imaging observation.

### Immunofluorescence assay, Prussian blue staining, and ICP-MS analysis of major organs

Gastric cancer tissues were harvested, embedded in OCT reagent, and cryosectioned at −20 °C by a cryostat (CM1900, Leica). Twenty micrometer-thick sections from representative areas were directly stained with 1 μg/mL PE-conjugated CD90 antibody for 20 min at room temperature; then, the specimen were rinsed with 0.1% PBS and examined by a fluorescence microscope (Olympus IX71). Tumor specimen intravenously injected FMNPs were used as the negative control. The slices were also stained with Prussian blue and nuclear fast red and visualized under a light microscope (Olympus IX71).

The iron content of gastric cancer tissues with FMNP-labeled MSCs were quantitatively detected by inductively coupled plasma mass spectrometry system (ICP-MS; Thermo Elemental X7, Thermo Scientific). Major organs such as the liver, heart, lung, kidney, and brain and the tumor were excised from the mice, cut into small pieces, weighed separately, and digested by nitric acid (67%, ultrapure reagent grade) and hydrogen peroxide (30%, ultrapure reagent grade). Then, the iron contents in these samples were quantified by ICP-MS.

### Hyperthermia therapy for nude mice with gastric cancer cells

Twenty nude mice loaded with gastric cancer were randomly divided into three groups: test group (10 mice, 5 × 10^6^ FMNP-labeled MSCs cells plus external magnetic fields), control group I (10 mice, 5 × 10^6^ FMNP-labeled MSCs cells ), and control group II (10 mice, FMNPs plus external magnetic fields). When the tumor size reached about 5 mm in diameter, nude mice were injected with FMNP-labeled MSCs and FMNPs via the tail vein, respectively. At 7 days post-injection, the mice in the test group and control group II were put under external alternating magnetic field with 63 kHz and 7 kA/m for 4 min [32] once a week for 1 month. The tumor sizes in the test group and control groups were measured every week.

### Analysis of chemokine receptors in MSC cells and chemokine in MFC cells

MSCs with positive CXCR4 and CCR7 were accounted by flow cytometer. FITC-conjugated CXCR4 antibody (BioLegend) and FITC-conjugated CCR7 antibody (BioLegend) were used to sort for MSCs with CXCR4- and CCR7-positive cells. The amounts of CXCL12 and CCL19 in the supernatants of MFC cells were examined by commercial enzyme-linked immunosorbent assay kits (R & D System, Shanghai, China). The migration ability of MSCs was evaluated by using a 48-well modified Boyden chamber [33-35]. The polycarbonate filter (12-μm pore size, CN110416, Neuroprobe, Bethesda, MD, USA) was pre-coated with 5 μg/mL fibronectin (Sigma). MSCs were resuspended at 5 × 10^5^/mL in the medium supplemented with 10% FBS and seeded in the upper chamber. Recombinant CXC ligand 12 (CXCL12; R&D System) and CCL19 (Peprotech, Rocky Hill, NJ, USA) were used as chemoattractants in the lower compartment. The chambers were incubated overnight at 37 °C. Results were expressed as the mean number of net migrated cells over control cells (basal migration without chemotactic stimulus), counted in ten microscope fields at high-power magnification (×1,000). Each experiment was performed in triplicate.

### Statistical analysis

All data are presented in this paper as mean result ± SD. Statistical differences were evaluated using the *t* test and considered significant at *P* < 0.05 level. All data in this article were obtained from three independent experiments.

## Results and discussion

### Identification of cultured MSCs

**Figure 1:**
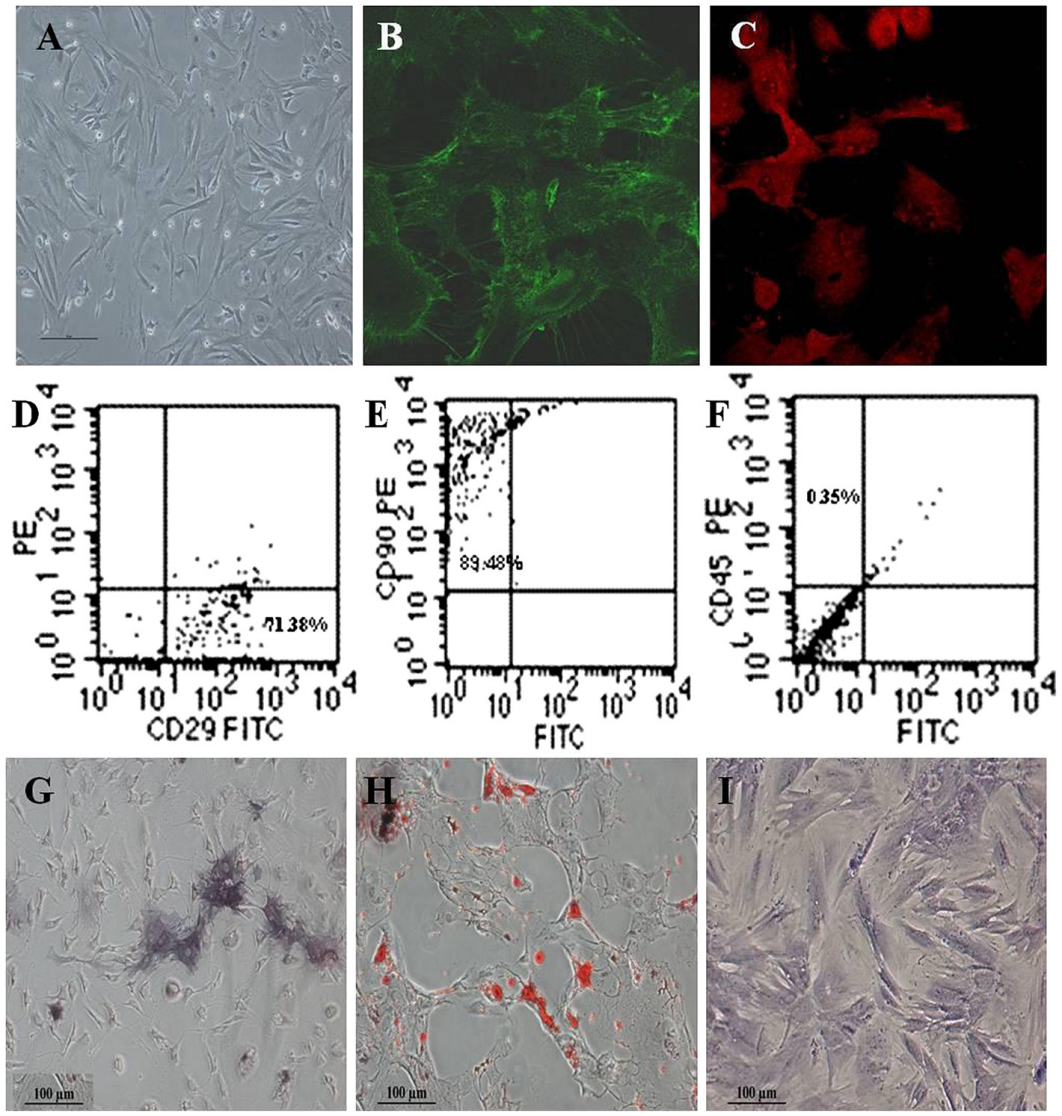
**Morphology and immunophenotypic characterization of MSCs.** (**A**) The fibroblastic morphology of passage 3 MSCs (magnification = ×100); (**B**) MSCs stained with FITC-conjugated CD29 antibody (×200); (**C**) MSCs stained with PE-conjugated CD90 antibody (×200); (**D**), (**E**), and (**F**) MSCs analyzed by FACS for the positive expression of CD29 (D) and CD90 (E) and negative expression of CD45 (F); (**G**) Differentiated osteoblasts tested with alkaline phosphatase staining (×100); (**H**) Differentiated adipocytes characterized by oil red O staining (×100); (**I**) Differentiated chondrocytes verified by toluidine blue staining (×100).

Mouse marrow MSCs were collected, cultured, and passaged for three passages and then identified by morphology. As shown in Figure 1a, MSCs grew out fibroblast-like shape. It is well known that MSCs own characteristic biomarkers, such as positively expressing adhesion molecules and stromal cell markers CD29 and CD90, and negatively expressing hematopoietic stem cell marker CD45. As shown in Figure 1b,c, immunofluorescence staining results showed that passage 3 MSCs positively expressed CD29 and CD90 antigens. FACS results showed that the expression rate of CD29 and CD90 in passage 4 MSCs were respectively 71.38% (Figure 1d) and 89.48% (Figure 1e), and the expression rate of CD45 was 0.35% (Figure 1f). MSC differentiation results showed that induced osteoblasts positively expressed alkaline phosphatase as shown in Figure 1g. The induced adipocytes had abundant intracellular oil-red-O-stained fat droplets as shown in Figure 1h. The differentiated chondrocytes formed a multilayered, matrix-rich morphology, and the extracellular matrix presented an aubergine color after being stained with toluidine blue as shown in Figure 1i. The above-mentioned results fully confirmed that prepared MSCs were multipotent stem cells.

### Characterization of FMNPs

**Figure 2:**
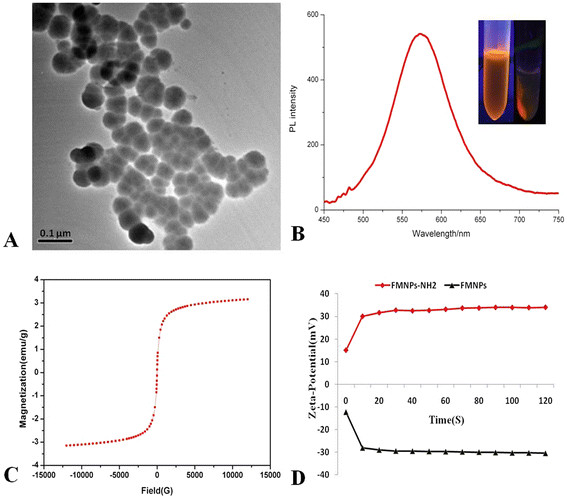
**Characterization of FMNPs.** (**A**) TEM image of amino-modified FMNPs; (**B**) PL intensity of amino-modified FMNPs; (**C**) Magnetic intensity curve of amino-modified FMNPs; (**D**) Zeta-potential of FMNPs with amino group and Si-O group.

The prepared FMNPs were encapsulated with CdTe quantum dots and Fe_3_O_4_ magnetic nanoparticles and modified with APS. Figure 2a is the TEM image of amino-modified FMNPs with the average diameter of 50 nm. Figure 2b shows the PL spectra of amino-modified FMNPs whose emission wavelength was 585 nm, and the inset is the fluorescent image of the prepared amino-modified FMNPs. Figure 2c shows that the prepared amino-modified FMNPs were superparamagnetic nanoparticles composed of Fe_3_O_4_ at room temperature with a saturation magnetization (*M*s) value of 3.2 emu·g^−1^. Figure 2d shows that prepared FMNPs had negative zeta-potential value of −30.80 mV, and amino-modified FMNPs had a positive zeta-potential value of 32.25 mV.

### Evaluation of FMNP-labeled MSCs

**Figure 3:**
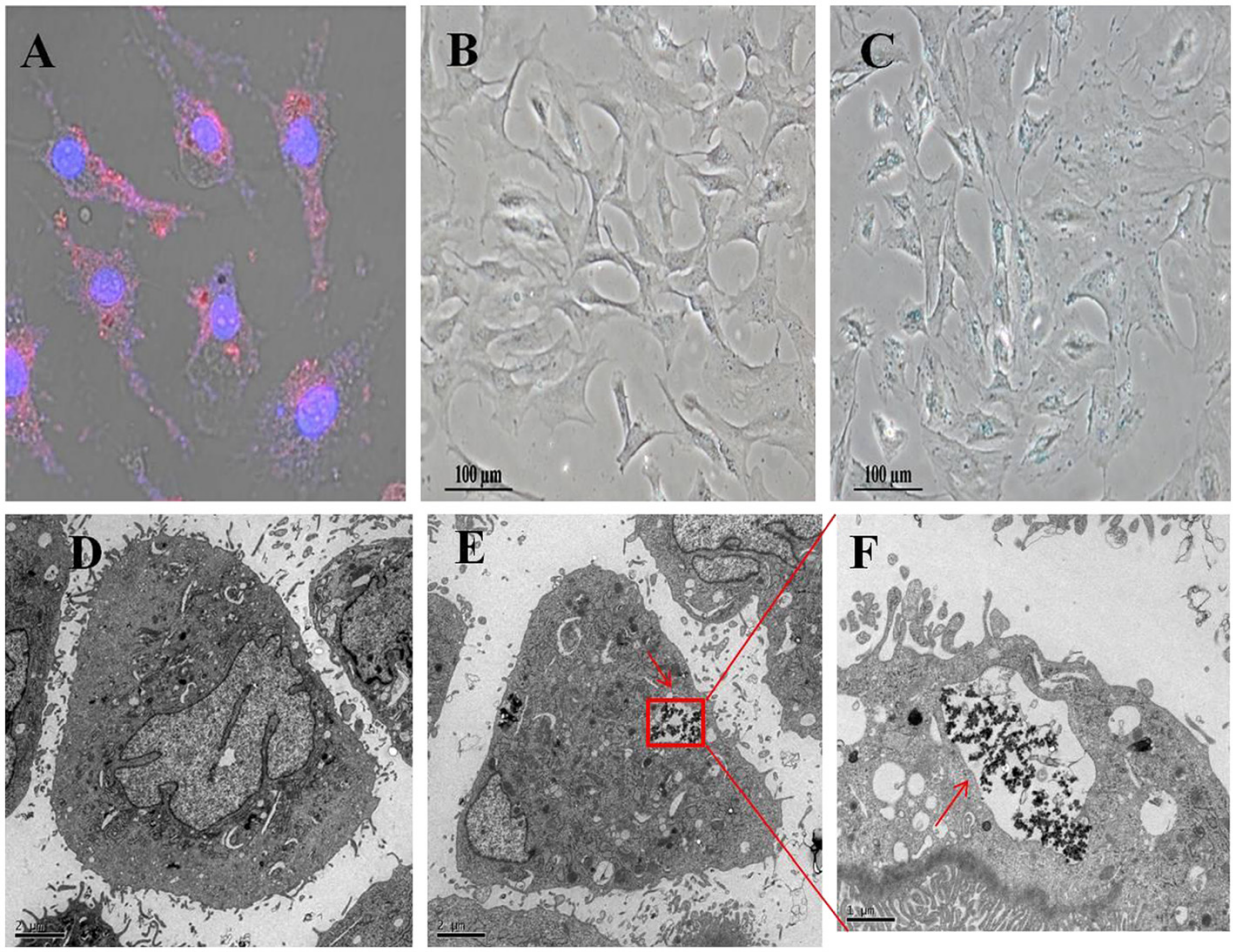
**Evaluation of FMNP-labeled MSCs.** (**A**) Cell nucleuses and FMNPs were visible in the MSCs by fluorescent microscope observation (×200); (**B**) FMNP-labeled MSCs were detected by using Prussian blue staining (×100); (**C**) Unlabeled MSCs were detected by using Prussian blue staining (×100); (**D**) The TEM image of unlabeled MSCs. (**E**) The TEM image of FMNP-labeled MSCs illustrates that amino-modified FMNPs were randomly distributed in the cytoplasm of MSCs (×6,000). (**F**) The magnification image of FMNPs distributed in the cytoplasm of MSCs (×12,000).

Cell membrane is negatively charged with phospholipid bilayer, and FMNPs with positive surface charges attached to the surface of the MSCs via electrostatic adsorption with higher efficiency compared with FMNPs with negative surface charges. As a result, amino-modified FMNPs were attached to and entered into *in vivo* MSCs, as shown in Figure 3a; fluorescent microscope images showed that amino-modified FMNPs located inside the cytoplasm of MSCs and emitted red fluorescence around the nucleus. Prussian blue staining results showed that some blue particles are located in the cytoplasm of FMNP-labeled MSCs (Figure 3c) and no blue signal was detected in unlabeled MSCs (Figure 3b). Furthermore, TEM results confirmed that FMNPs located inside MSCs (Figure 3e,f) and the labeled MSCs had a distinct appearance compared to the unlabeled MSCs as shown in Figure 3d.

**Figure 4:**
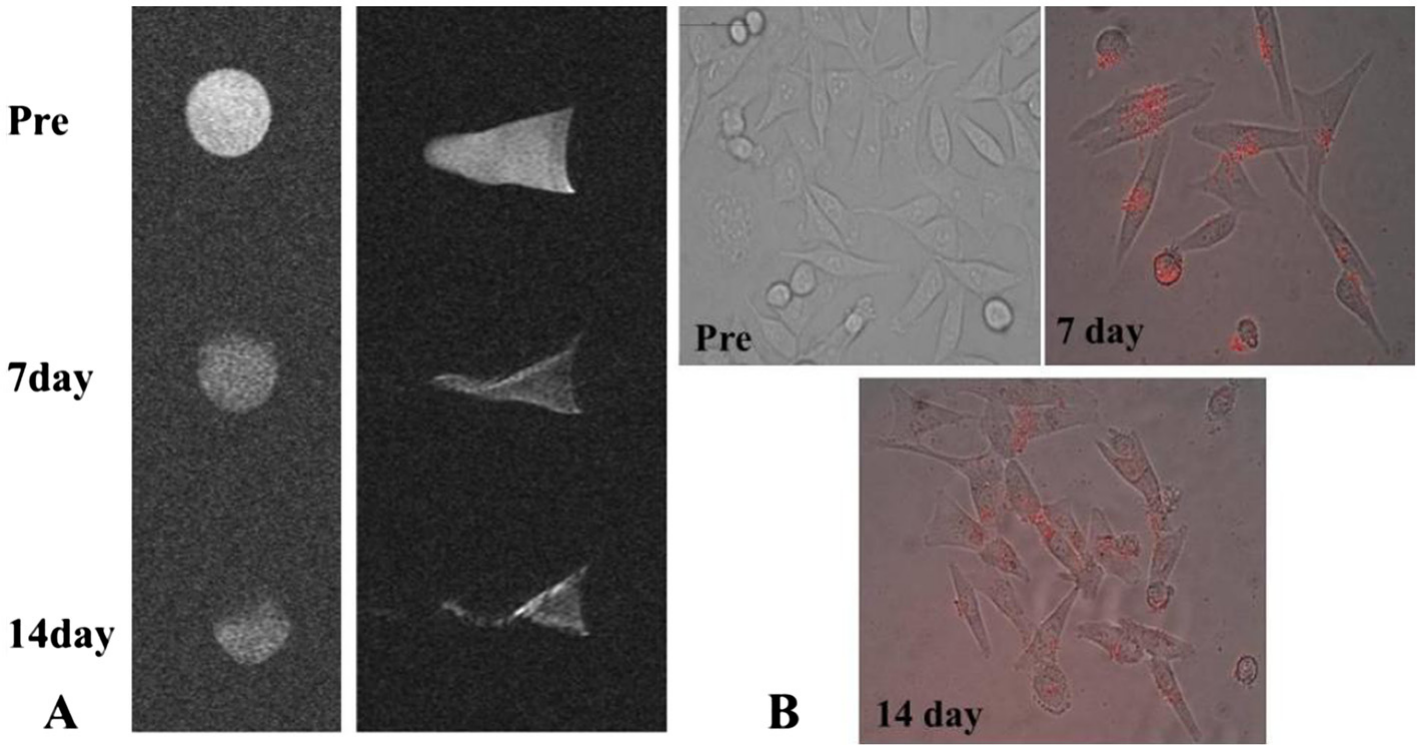
**Label stability of the FMNP-labeled MSCs.** (**A**) MR imaging of FMNP-labeled MSCs at 7 and 14 days. (**B**) Fluorescent microscope images of FMNP-labeled MSCs at 7 and 14 days (×200).

In order to confirm that FMNP-labeled MSCs have stable fluorescent signal and magnetic resonance performances up to 14 days, unlabeled MSCs and FMNP-labeled MSCs were respectively examined with a fluorescent microscope and MR imaging system at 7 and 14 days, as shown in Figure 4a. FMNP-labeled MSCs emitted red strong fluorescence signal in the cytoplasm compared to the unlabeled MSCs; the labeled MSCs at 7 and 14 days had stable fluorescence signal, and no fluorescence quenching was observed, similar to other reports [36,37]. The similar results were exhibited in the MR images as shown in Figure 4b. Labeled MSCs had higher magnetic resonance signals than unlabeled MSCs, and labeled MSCs at 7 and 14 days still had similar magnetic resonance intensity. These results showed that labeled MSCs kept labeled status up to 14 days.

### Cytotoxicity of amino-modified FMNPs

**Figure 5:**
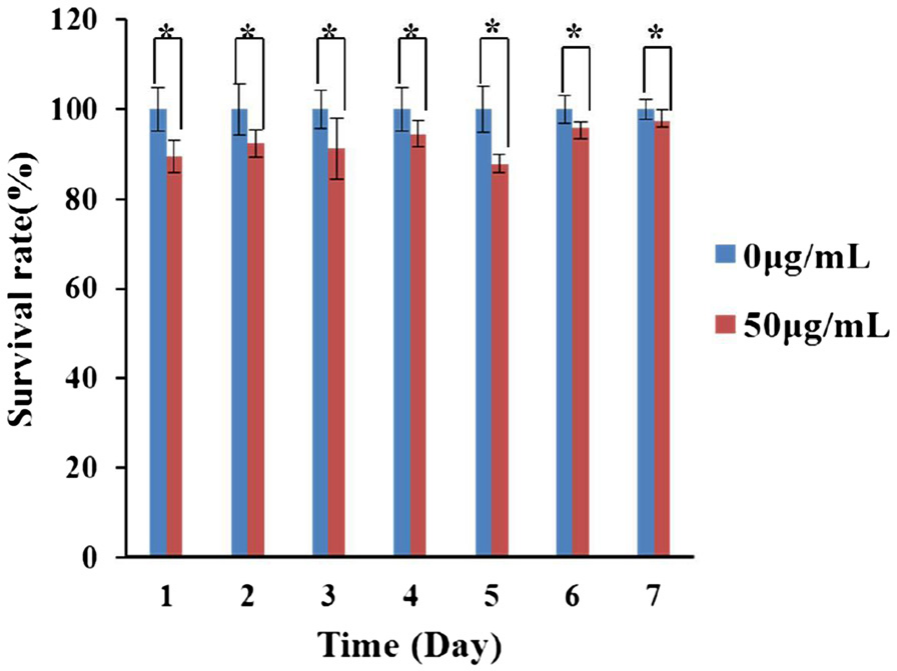
**Effects of amino-modified FMNPs on MSCs.** The survival rates of MSCs after being labeled with FMNPs keep increasing from day 1 to day 7. Mean values ± standard deviation of independent experiments are shown. Asterisk represents *P* < 0.05

As shown in Figure 5, amino-modified FMNPs under the dose of 50 μg/mL did not exhibit obvious cytotoxicity to MSCs, and the survival rate of MSCs kept increasing within 7 days; no statistical difference existed between FMNP-labeled MSCs and pure MSCs (*P* < 0.05). This result was similar to our previous report [38], which implied that amino-modified FMNPs had good biocompatibility to MSCs within the dose of 50 μg/mL and to nude mouse within the dose of 2 mg/Kg body weight within 30 days.

### FMNP-labeled MSCs for targeted imaging of gastric cancer cells *in vivo*

**Figure 6:**
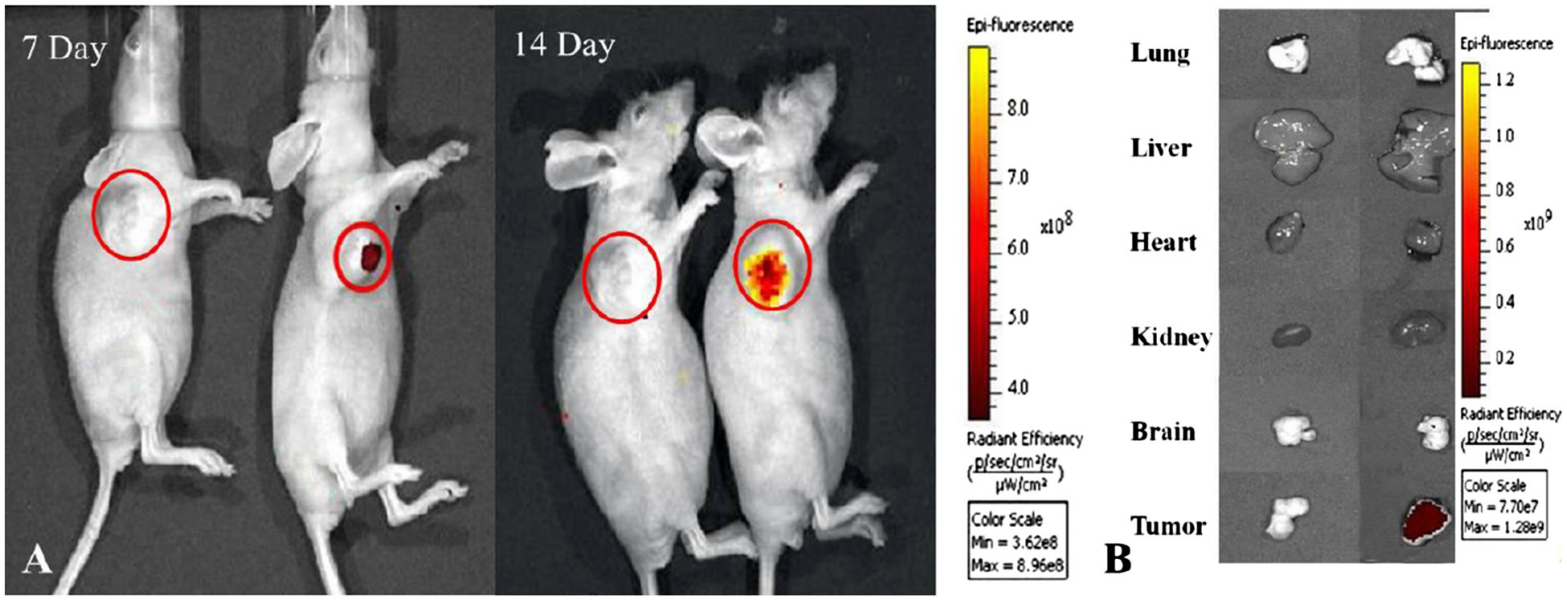
**Fluorescent imaging of FMNP-labeled MSCs targeting gastric cancer cells*in vivo.*** (**A**) The *in vivo* fluorescent images show that tumor sites of the mice in the test group had fluorescent signals after post-injection of FMNP-labeled MSCs at 7 and 14 days (right), and tumor sites of the mice in the control group had no fluorescent signal after post-injection of FMNPs at 7 and 14 days (left). (**B**) The fluorescent imaging of major organs show that no signal was detected in the tumor and organs of the control group (left), and obviously fluorescent signals were detected in the tumor tissues of the test group (right).

The nude mouse models loaded with gastric cancer tissue with a diameter of approximately 5 mm were successfully prepared. The FMNP-labeled MSCs were intravenously injected into gastric cancer mouse models their distribution in the whole body was monitored for 14 days post-injection by using IVIS fluorescence imaging system and MRI instrument. As depicted in Figure 6a, no signal was detected in the tumor tissues in the control group (left), while the fluorescence signals were clearly detected in tumor tissue in the test group (right), and the fluorescent signals in tumor tissues gradually increased from day 7 to 14 post-injection. At day 14 post-injection, major organs, such as the lungs, kidneys, hearts, livers, and brains, and the tumor tissues were collected from euthanatized mice and imaged by using IVIS fluorescence imaging system. Tumor tissues in the test group had remarkably fluorescent signals compared to those in the control group, as shown in Figure 6b, and there was no specific fluorescent signal detected in the heart, lung, kidney, brain, and liver.

**Figure 7:**
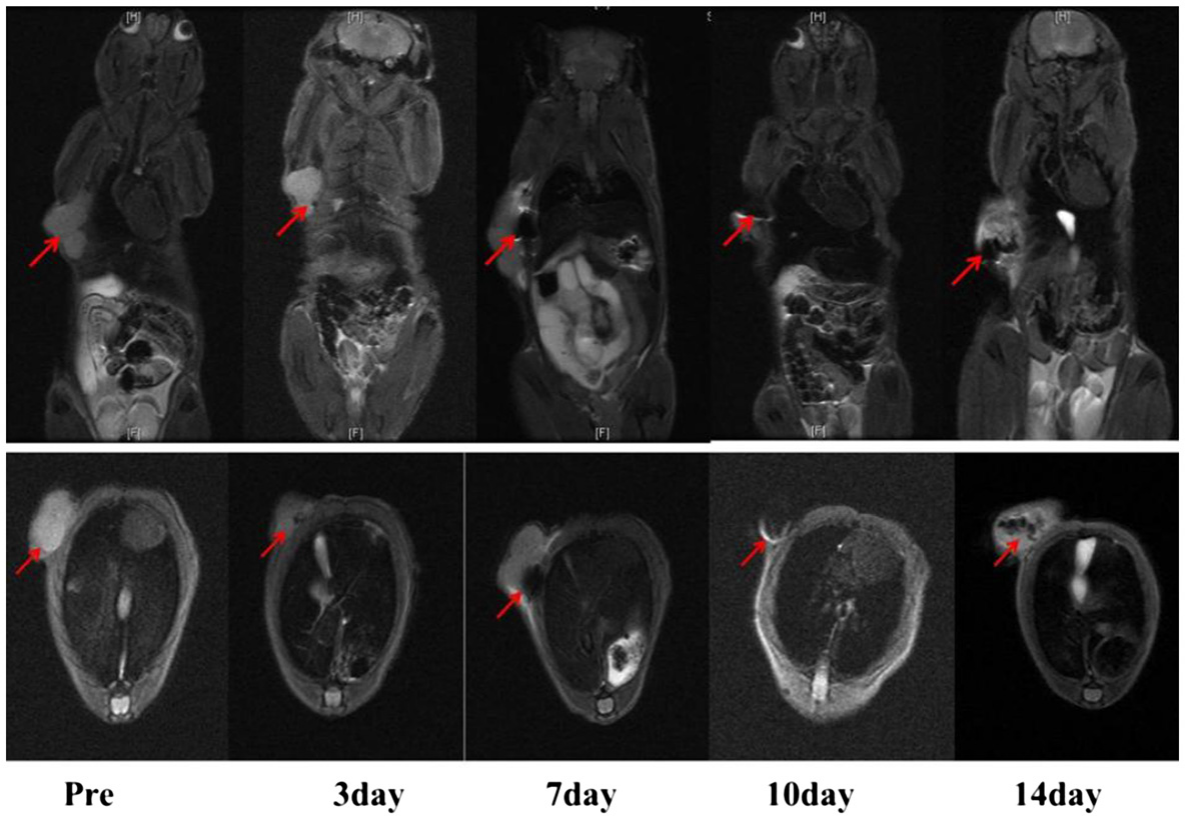
**MR imaging of FMNP-labeled MSCs targeting gastric cancer cells.** The coronal and transected images of MRI show that MSCs firstly presented in the tumor tissue at 7 days post-injection and then gradually accumulated in the tumor tissue and reached peak value at 14 days post-injection.

Furthermore, the targeting ability of labeled MSCs in the gastric cancer mouse models were monitored by MR imaging system at 3, 7, 10, and 14 days post-injection, as shown in Figure 7; the coronal and transected images of the MRI showed that MSCs firstly presented in the tumor tissue at day 7 post-injection and then gradually accumulated in the tumor tissue and reached peak value at day 14 post-injection. Actually, the migration of MSCs to the tumor is a gradual course; MSCs preferentially migrated to the tumor area and released abundant chemokines into microenvironment and then accumulated around the tumors by EPR. The significant magnetic resonance signals from the surrounding of the tumors were also observed at 7 days post-injection, and the magnetic resonance signals inside the tumors were gradually increased at 14 days post-injection. The above-mentioned results fully demonstrate that FMNP-labeled MSCs could target and migrate to the gastric cancer cells *in vivo*.

### Immunofluorescence staining, Prussian blue staining, and ICP-MS analysis of major organs

**Figure 8:**
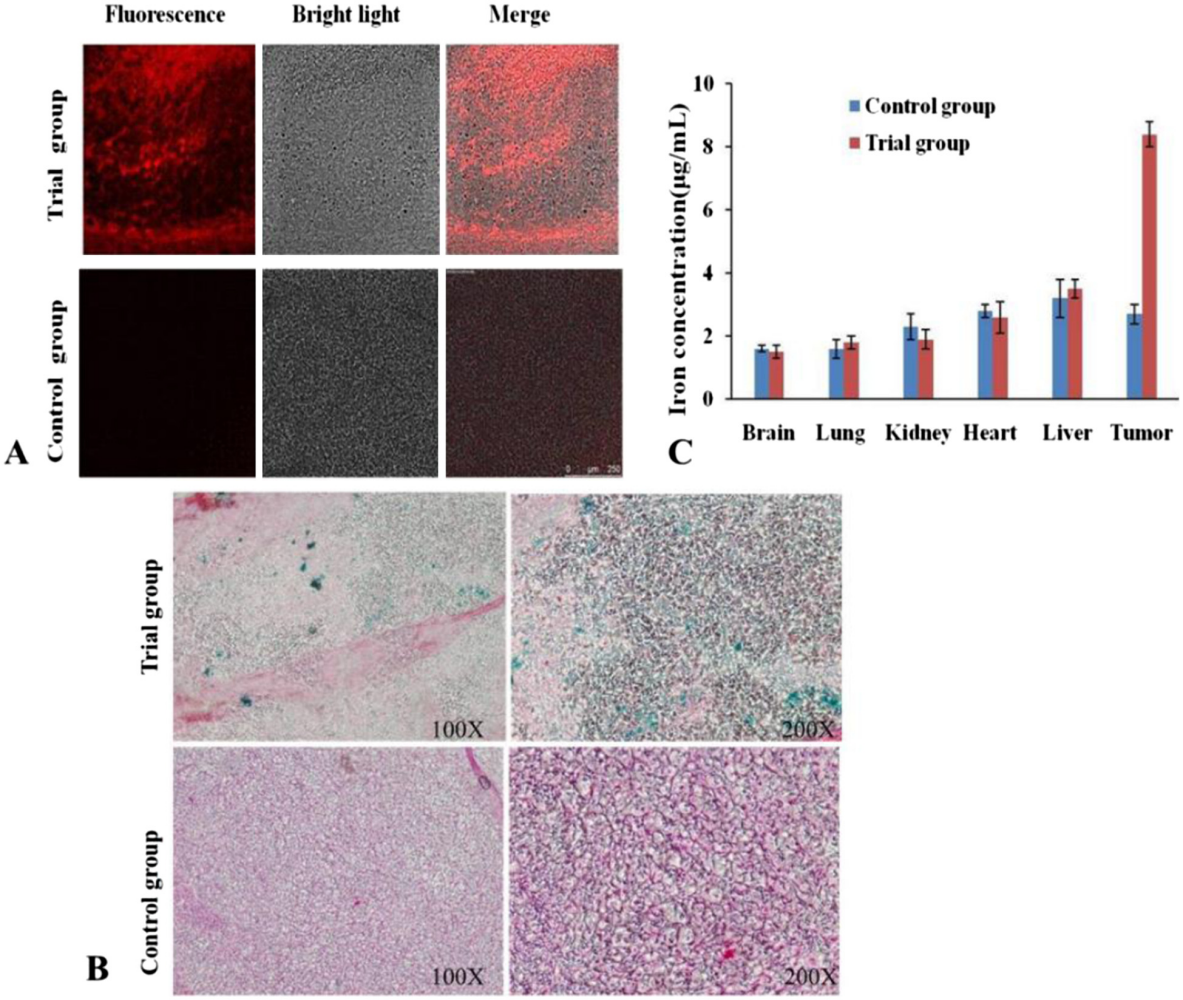
***Ex vivo*analysis of major tissues.** (**A**) Immunofluorescence-staining analysis shows that the tumor tissue slices in the test group had obvious fluorescent signals and that there was no fluorescent signal in the control group. (**B**) Prussian blue-staining results show that the tumor tissue slices in the test group had blue iron particles, and that there was no iron particle in the control group. (**C**) The ICP-MS results show that FMNPs were mainly distributed in the tumor tissues of the test group.

In order to further confirm the FMNP-labeled MSCs located in the tumor tissue, immunofluorescence staining and Prussian blue staining of the tumor tissues were performed. The tumor tissues were collected, and ultra-thin tumor slices were prepared and stained with PE-conjugated anti-CD90 antibody. As shown in Figure 8a, the tumor tissues in the test group had distinct red fluorescent signals; conversely, the tumor tissues in the control group had no fluorescent signal. The prepared ultra-thin tumor slices were also treated with Prussian blue staining to validate the labeled MSCs distributed in the tumor tissues, as shown in Figure 8b; tumor tissues in the test group had significant blue iron particles, and the tumor tissues in the control group had no blue signal. These results fully indicate that the FMNP-labeled MSCs could target the gastric cancer tissues.

The distribution of labeled MSCs *in vivo* was also analyzed by ICP-MS. The concentration of iron in the tumor tissues of the test group (8.4 μg/mg) was significantly higher than that of the control group (2.7 μg/mg), as shown in Figure 8c. For other organs such as the liver, heart, lung, brain, and kidney, no statistical difference was observed between the test group and the control group (*P* < 0.05). These data also demonstrate that FMNP-labeled MSCs mainly distribute in the site of gastric cancer tissues *in vivo*.

### Hyperthermia therapy of subcutaneous tumors

**Figure 9:**
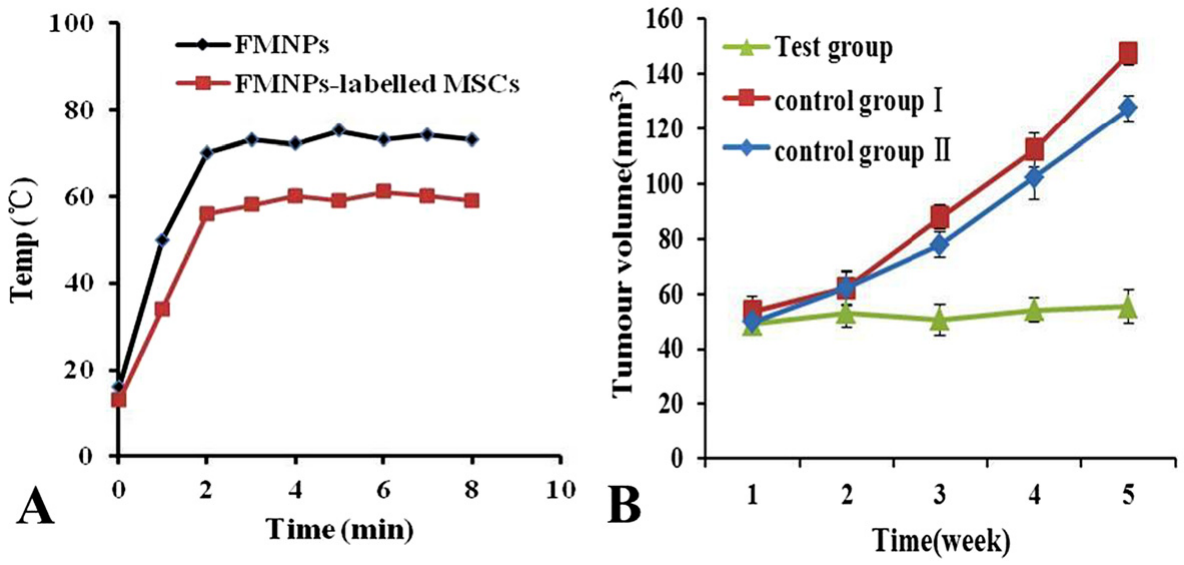
**Effects of alternating magnetic field on the gastric tumor size.** (**A**) Time-temperature curve of FMNP-labeled MSCs under an alternating magnetic field with 63 kHz and 7kA/m; (**B**) The growth of tumors was inhibited under hyperthermia therapy.

The nude mice loaded with gastric cancer in the test group were injected with FMNP-labeled MSCs, the nude mice loaded with gastric cancer in control group I were also injected with FMNP-labeled MSCs, and the nude mice loaded with gastric cancer in control group II were injected with FMNPs. At 7 days post-injection, the nude mice in the test group and control group II were treated with alternating magnetic field with 63 kHz and 7 kA/m for 4 min once a week for 1 month, and the nude mice in control group I were not treated. Figure 9a shows the time-temperature curves of FMNPs and FMNP-labeled MSCs under alternating magnetic field irradiation, which indicate that the response temperature of FMNP-labeled MSCs is lower than that of FMNPs. As shown in Figure 9b, the average size of tumors in the test group is significantly smaller than in control group I and control group II (*P* < 0.05), and the average size of tumor in control group I was bigger than in control group II, which demonstrates that FMNP-labeled MSCs may not inhibit the growth of tumor. However, FMNP-labeled MSCs combined with alternating external magnetic field could markedly inhibit the growth of tumor cells *in vivo*.

### Possible molecular mechanism

**Figure 10:**
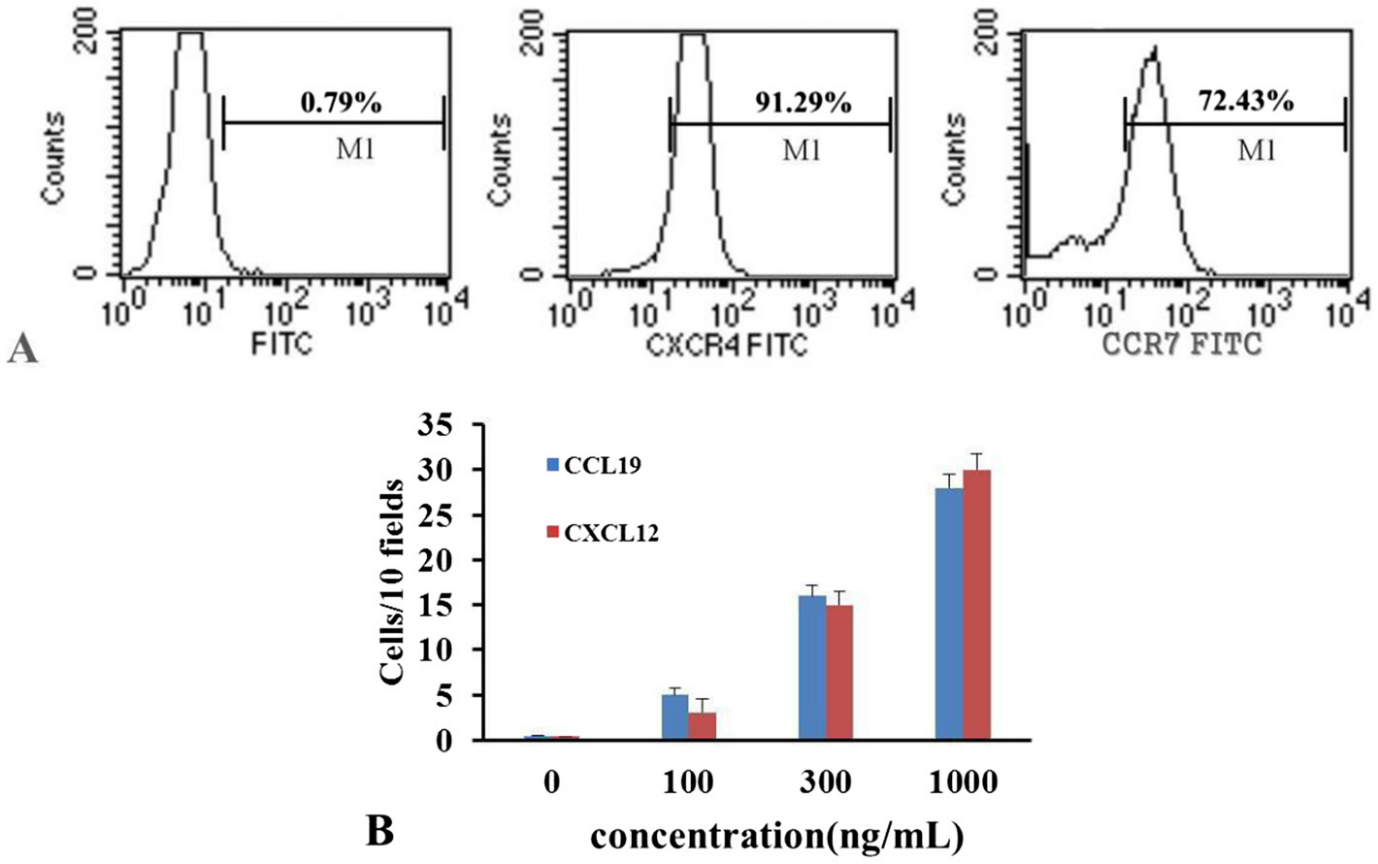
**Analysis of chemokine receptors and chemotaxis.** (**A**) The expressions of CXCR4 and CCR7 on MSCs were determined by FACS. (**B**) The migration of MSCs to different concentrations of CXCL12 and CCL19 was evaluated by chemotaxis assay.

Since chemokines and chemokine receptors are involved in chemotaxis and cell migration, in order to investigate the mechanism of FMNP-labeled MSCs migrating to gastric cancer sites *in vivo*, we analyzed the chemokine receptors' expression levels in MSCs and measured the chemokine levels in the supernatant of the MFC cells. As shown in Figure 10a, flow cytometer analysis showed that 91.29% of mES cells exhibited positive CXCR4 and that 72.43% of mES cells exhibited positive CCR7. ELISA results showed that CXCL12 and CCL19 levels in the supernatant of MFC cells were 13.2 ± 1.8 ng/mL and 5.5 ± 0.7 ng/mL, respectively. Then, the migration ability of MSCs in response to chemotactic signals was investigated by using a micro-multiwell chemotaxis chamber assay. Results show that MSCs migrated to different concentrations of CXCL12 and CCL19 in a dose-dependent manner, as shown in Figure 10b. Therefore, in the course of MSCs targeting gastric cancer cells *in vivo*, the loops such as CXCL12-CXCR4 and CCL19-CCR7 may take great effects. Although our primary studies show that mouse MSCs can target *in vivo* mouse gastric cancer cells and also confirm that CXCL12-CXCR4 and CCL19-CCR7 loops were involved in the course, the concrete mechanism of the mouse MSCs targeting gastric cancer cells *in vivo* is still under investigation.

## Conclusion

In conclusion, this study clearly confirms that FMNP-labeled MSCs could target gastric cancer cells *in vivo* and could be used as dual-modality contrast agents for *in vivo* gastric cancer's fluorescent imaging and magnetic resonance imaging. The prepared FMNPs had good biocompatibility and could be used for hyperthermia therapy of gastric cancer *in vivo* under a given external magnetic field. Regarding the mechanism of FMNP-labeled MSCs recognizing *in vivo* gastric cancer cells, we consider that CCL19/CCR7 and CXCL12/CXCR4 axis loops may be involved in this course. The concrete mechanism is under investigation. Therefore, FMNP-labeled MSCs have great potentials in applications such as detection, fluorescent imaging, magnetic resonance imaging, and simultaneous hyperthermia therapy for early gastric cancer in the near future.

## Competing interests

The authors declare that they have no competing interests.

## Authors' contribution

JR and JJ synthesized and characterized the materials, finished most of the cellular and mice experiments, and drafted the manuscript. HS carried out partial bio-safety study. KW performed the statistical analysis. CW participated in the mice experiments. QQ and DC conceived of the study, participated in its design and coordination and in revising the manuscript. All authors read and approved the final manuscript.
